# 4553 An investigation into the use of curcumin, a topical herbal agent, for the treatment of cervical intraepithelial neoplasia

**DOI:** 10.1017/cts.2020.368

**Published:** 2020-07-29

**Authors:** Emily Wang, Theresa Kuhn, Cecile Lahiri, Minh Nguyen, Ighovwerha Ofotokun, Rachael Abraham, Kirk Easley, Marina Mosunjac, Talaat Tadros, Catherine Finneran, Lisa Flowers

**Affiliations:** 1 Morehouse School of Medicine; 2 Emory University School of Medicine; 3 Emory University Rollins School of Public Health

## Abstract

OBJECTIVES/GOALS: Cervical cancer is the fourth most common cancer among women worldwide, with approximately 570,000 newly diagnosed cases and 311,000 related deaths among women in 2018.In the United States, approximately 13,000 new cases of cervical cancer are diagnosed annually with approximately 4,000 women dying each year from cervical cancer. Nearly all cervical cancer is caused by oncogenic strains of the human papillomavirus (HPV). Prevention strategies to reduce cervical cancer after HPV exposure entail treatment of cervical dysplasia at the premalignant state, specifically low-grade squamous intraepithelial lesions (LSIL) and high-grade squamous intraepithelial lesions (HSIL), along with the eventual clearance of HPV.The most common treatment for persistent LSIL or HSIL is the loop electrical excisional procedure (LEEP). This procedure is unfortunately not widely available in resource-limited countries and is associated with potential significant morbidities, including decreased fertility, preterm birth, premature rupture of membranes and cervical incompetence. Despite undergoing standard of care treatment, in certain high-risk populations, specifically HIV-infected women, there is a higher rate of premalignant HPV-related cervical disease persistence, progression and recurrence.There is a paramount need for novel nonsurgical treatments to stabilize or treat precancerous lesions of the cervix and to decrease the persistence of HPV infection. Medical treatment with the natural herb curcumin may allow subjects to receive treatment of cervical lesions without undergoing a surgical procedure.Curcumin is a major active component extracted from turmeric with anti-inflammatory, anti-infectious, and anti-cancer properties.Topical intravaginal curcumin has the promise of delivering this drug directly to the site of disease, ensuring adequate concentrations at the site of disease while avoiding systemic side effects. This proposed study will determine if there are higher rates of HPV clearance after curcumin administration among women with and without HIV who have premalignant HPV-related cervical disease, specifically LSIL or recently treated (with a LEEP) HSIL. METHODS/STUDY POPULATION: We are proposing a prospective double-blind randomized control trial to investigate the utility of topical intra-vaginal curcumin in increasing rates of HPV clearance and mitigating the high rates of disease recurrence in women with and without HIV. A sample of approximately 200 women with and without HIV who have biopsy-proven LSIL or recently treated HSIL will be randomized to one of two arms: 2000 mg of curcumin powder in capsules or placebo inserted intravaginally at bedtime once weekly for 20 weeks (excluding the time of menses). Cervical cytology and HPV testing will be performed at baseline and 6 months post-randomization. If a participant has abnormal cytology or a positive high-risk HPV test 6 months post-randomization, they will be scheduled to undergo a colposcopy with biopsies of all suspicious cervical lesions. If biopsy results are HSIL or greater, subjects will be referred back to routine clinical treatment, which may include a LEEP. Clinicians performing the colposcopies and the study participants will be blinded since the placebo has the same appearance as the curcumin capsules. At the end of the study, study participants will be offered the opportunity to participate in 2-3 hour focus groups to discuss acceptability of the product as a treatment for premalignant HPV-related cervical disease until data saturation is achieved. Power and sample size calculations are based on the primary outcome of interest, which is clearance of HPV at 6 months. Basu et al (2013) documented HPV clearance in as many as 80% of subjects with topical curcumin. To account for an expected lower success rate in HIV-infected women, who will also be included in the study, we intend to power our study to determine a more conservative 20% improvement in clearance rate at 6 months with curcumin treatment, assuming an expected clearance rate of HPV in HIV-infected women of 25%. In order to detect a 20% difference of HPV clearance among those treated with intravaginal curcumin vs. placebo at 6 months, about 80 patients per arm would achieve 80% power at the 5% significance level. To account for up to 20% loss to follow-up or discontinuation, the total sample size in each arm would be 100 subjects with a total of approximately 200 subjects enrolled in both arms. RESULTS/ANTICIPATED RESULTS: We are currently in the process of collecting data for this study. We hypothesize that intravaginal curcumin will have a 20% higher rate of HPV clearance at 6 months as compared to placebo. Primary outcome measures will include clearance of HPV at 6 months in curcumin vs. placebo. Secondary outcomes measures will include recurrence of disease by either cytologic or histologic abnormality requiring further surveillance or treatment at 6 months. We also hypothesize that intravaginal curcumin administered once weekly at bedtime for 20 weeks will be safe, acceptable, and well tolerated. This is based off of previous findings from the Phase 1 trial of intravaginal curcumin that we performed. During this Phase 1 trial, we explored daily intravaginal administration of 2000 mg of curcumin to further understand curcumin’s tolerability. Our focus group participants displayed an overwhelming consensus that daily administration affected quality of life, specifically due to the yellow-colored vaginal discharge from the medication. Study participants expressed that once or twice weekly administration was more tolerable and feasible. Our proposed study would therefore test the tolerability and effects of weekly curcumin administration and its ability to clear HPV infection. The primary outcome measure will be the proportion of study participants who discontinue treatment for any reason (acceptability) and the proportion of study participants who discontinue treatment due to adverse effects (tolerability). DISCUSSION/SIGNIFICANCE OF IMPACT: Non-surgical treatments that decrease the morbidity and risk of progression of premalignant HPV-related cervical disease are greatly needed, especially in low-resource settings and among women experiencing barriers to care and/or at high risk for disease progression. Medical treatment with the natural herb curcumin is an emerging strategy that may allow subjects to receive treatment of cervical lesions without undergoing a surgical procedure.Several preclinical and clinical studies have shown curcumin’s ability to reduce tumors and precancerous lesions in animal and human cancer cells. Curcumin can suppress the activation of transcription factor NF-κB and the expression and activity of VEGF and p16INK4a, biomarkers known to be elevated in premalignant HPV-related cervical disease.Studies have also shown that curcumin alters HPV-associated molecular pathways in cancer cells, suppressing cervical cancer growth by inhibiting the transcription of oncoproteins HPV16 and HPV18 (designated as E6 and E7) and restoring p53 and retinoblastoma function. Our proposed study would therefore test the tolerability and effects of weekly curcumin administration and its ability to clear HPV infection. Our results will generate novel data as to what is an acceptable and well-tolerated dosing regimen of intravaginal curcumin, which would be crucial in designing further curcumin intervention studies. The results of our proposal will explore the effect of intravaginal curcumin as a standalone and adjuvant therapy to a LEEP among women with premalignant HPV-related cervical disease. The potential to not just excise diseased tissue, but to directly augment the clearance of the causative agent HPV, would have profound long-term ramifications in resource-limited settings and among women experiencing barriers to care and/or at high risk for disease progression.

